# A Dangerous Hiding Spot: The Unrecognized Danger Posed by Child-Sized Helium Balloons

**DOI:** 10.7759/cureus.73168

**Published:** 2024-11-06

**Authors:** Katherine Ritter, Tania Ahluwalia

**Affiliations:** 1 Pediatric Emergency Medicine, Children's National Medical Center, Washington DC, USA

**Keywords:** anticipatory guidance, asphyxia, child injury prevention, pediatric emergency and acute care, pediatric emergency department (ped), suffocation

## Abstract

Anticipatory guidance on balloons typically highlights the danger of choking on uninflated balloon fragments. One type of balloon injury that is not widely discussed is suffocation due to crawling inside a large foil helium-containing balloon. A six-year-old female presented to a community hospital emergency department (ED) after being found on the floor inside a 50-inch foil balloon in the shape of the number “7.” The patient’s mother found her unconscious, limp, and with no spontaneous respirations. She initiated chest compressions before emergency medical services arrived. After evaluation and treatment in the community hospital ED, the patient was transferred to a tertiary children’s hospital for observation and frequent neurological checks. She returned fully to baseline mental status 12 hours after the injury and was discharged home later that same day. This case highlights that oversized balloons can be enticing but hazardous hiding spots for children and discusses several possible mechanisms of injury. Parents and pediatricians should be aware of the dangers of suffocation in these cases.

## Introduction

Balloons are the leading cause of suffocation death among children’s toys [1]. The U.S. Consumer Product Safety Commission warns that balloons pose a significant suffocation risk if unintentionally swallowed or inhaled and warns against children under the age of eight playing with uninflated balloons.

There is also the risk of helium toxicity. Helium inhalation causes hypoxia by displacing oxygen in the lungs [2]. The National Electronic Injury Surveillance System database reports a higher prevalence of helium inhalation injuries from 2000 to 2019, most commonly in males aged 12 and younger [3]. The most common symptoms of helium inhalation were syncope, head injury, dizziness, contusion/abrasion, and concussion. In 98.3% of cases, the patients were discharged home from the emergency department (ED) [3].

There is a paucity of literature on balloon injuries from mechanisms other than choking on uninflated balloons or intentionally inhaling helium. However, there are examples in news articles. In 2006, college students were found dead inside an eight-foot deflated helium-containing promotional balloon after apparently crawling inside it [4]. The deaths were presumed to result from suffocation by helium inhalation [5]. In 2016, an eight-year-old was found dead with a large foil balloon over her head. Her parents hypothesized that she had opened the balloon, became trapped inside, and suffocated [6]. Similarly, in 2021, an eight-year-old died after entering an “8"-shaped balloon [7]. He was found unresponsive, with the balloon completely covering his head. In this case, the balloon was one week old and partially deflated, but still floating. Finally, in 2022, a five-year-old died after climbing inside a large dinosaur-shaped balloon [8]. His parents hypothesized that he entered the balloon because he had been trying to dress up as a dinosaur. This case report describes the case of a six-year-old who was found down after crawling inside a 50-inch foil balloon and discusses several possible mechanisms of injury.

This article was previously presented as a meeting abstract at Children's National Research, Education, and Innovation Week on April 23, 2024, in Washington, DC.

## Case presentation

**Figure 1:**
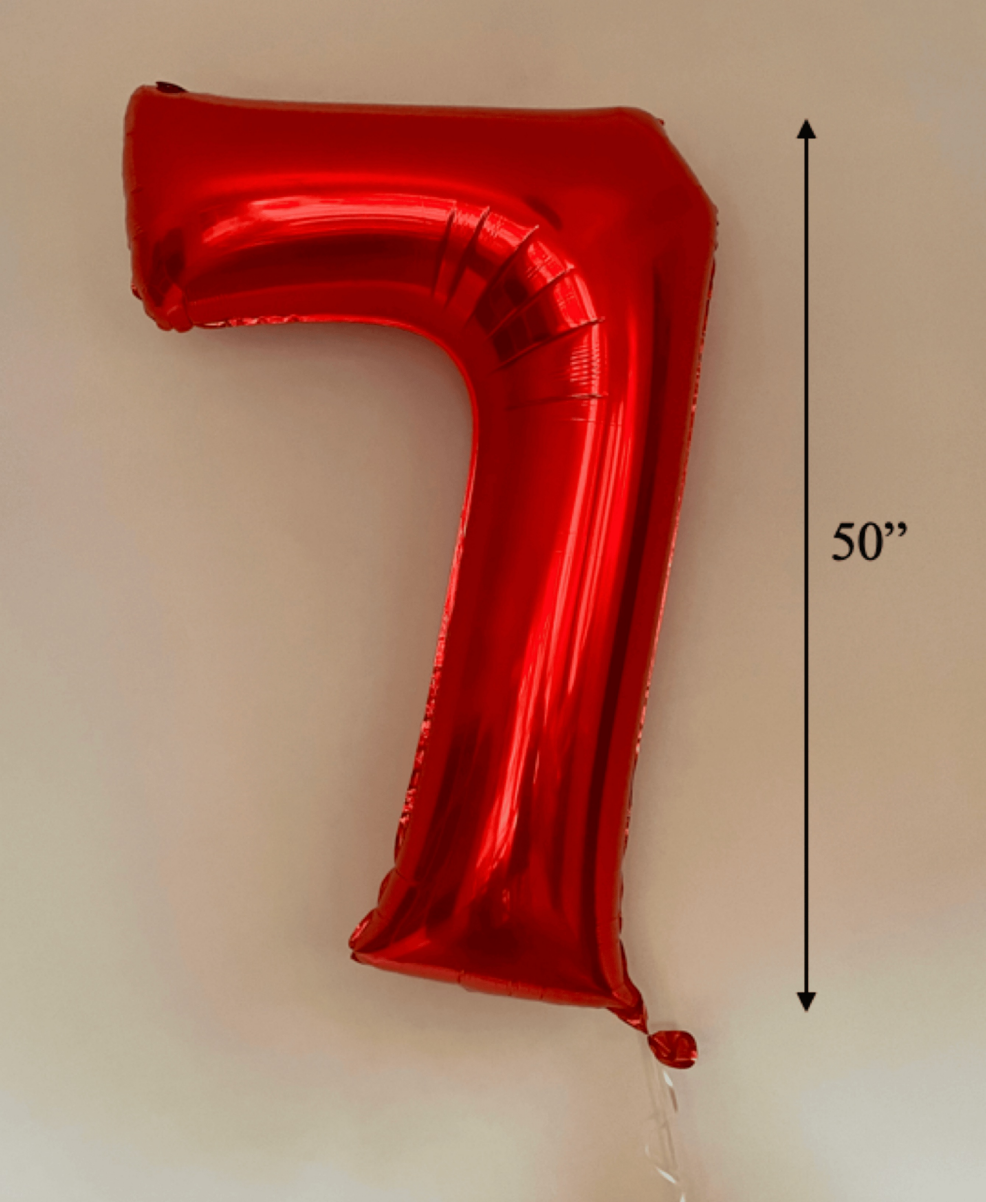
Image of a balloon similar to the balloon the patient was found inside

A previously healthy, developmentally normal six-year-old female presented to a community hospital ED via emergency medical services (EMS) after being found down for an unknown period of time inside a 50-inch foil balloon in the shape of the number “7." According to the patient’s mother, the patient had been playing alone indoors with both traditional balloons and two 50-inch, floating, helium-containing foil “7” balloons (Figure 1).

**Figure 2:**
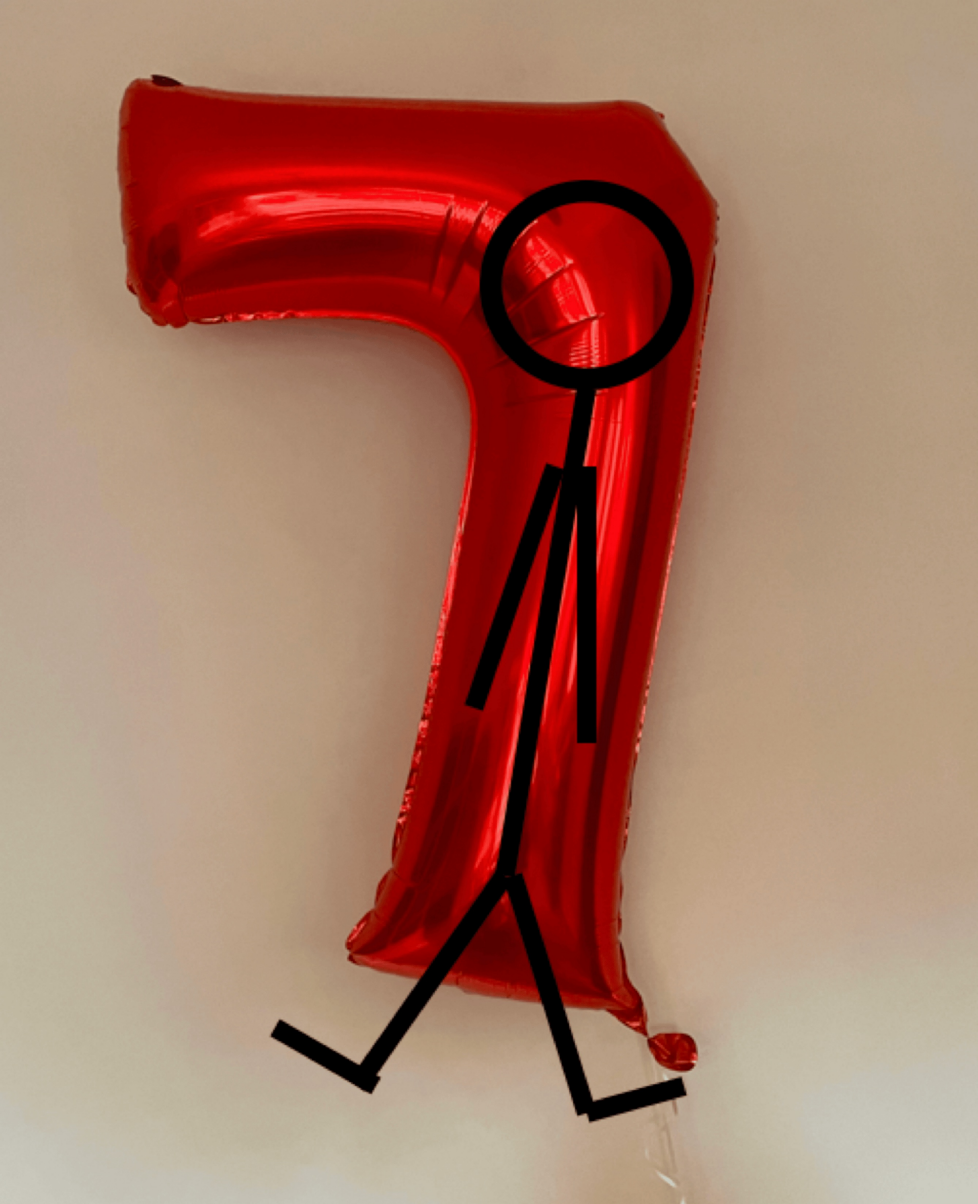
Diagram illustrating how the patient was found inside the balloon

The patient's mother found the patient inside one of the large foil "7" balloons. The patient had opened up the balloon and was found inside, with her head in the bend of the balloon, her body inside the long part of the “7” with her arms by her sides, and only her feet and ankles visible outside the balloon (Figure 2). The patient was unconscious, limp, and with no spontaneous respirations. It is not known how long the patient was inside the balloon or how long the patient was unconscious.

**Figure 3:**
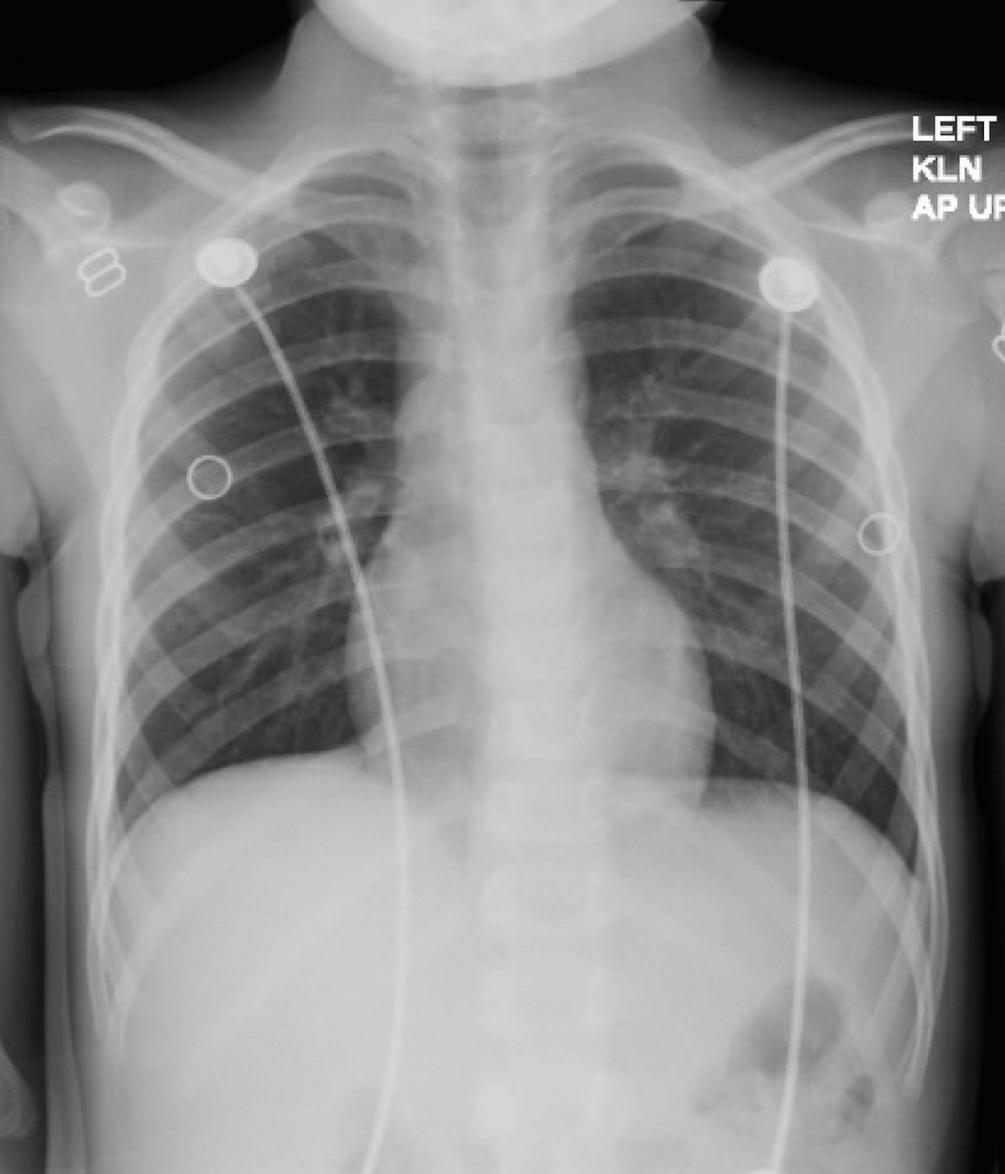
Patient's chest radiograph

**Figure 4:**
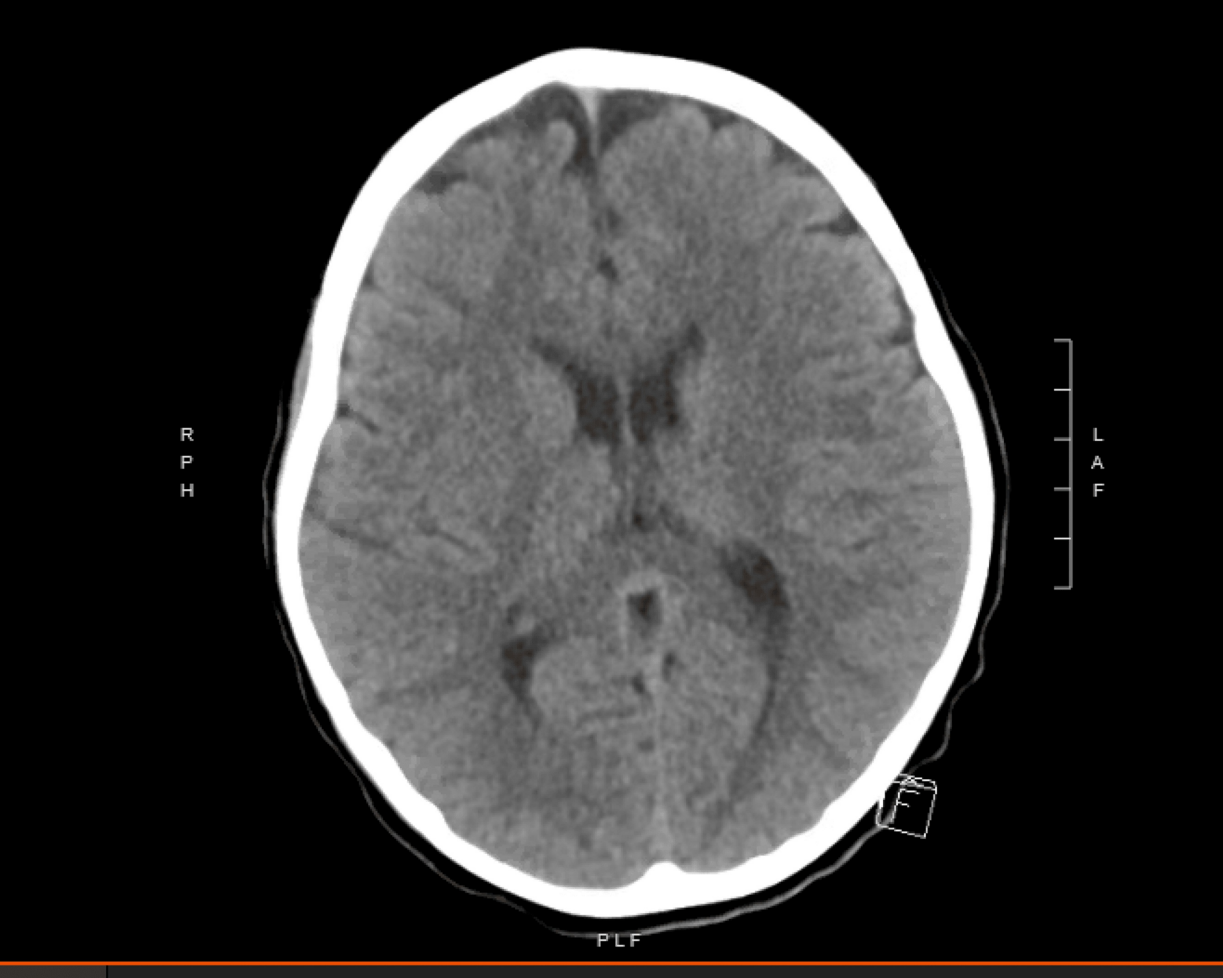
Patient's head CT CT: computed tomography

The patient’s mother and a neighbor initiated chest compressions. They did not check for a pulse before starting compressions. After approximately four minutes of compressions and rescue breaths, the patient began taking spontaneous respirations. When EMS arrived, the patient was conscious and breathing spontaneously but was not oriented. Her initial vitals were a heart rate of 120 beats per minute and blood pressure of 127/86 mmHg. She was placed on oxygen via nasal cannula by EMS. She had an episode of urinary incontinence but no abnormal body movements such as stiffening, shaking, clonus, or tongue biting. On arrival at a community ED, she was drowsy but alert and oriented. She had an episode of non-bloody and non-bilious emesis in the ED. Her initial vitals included a temperature of 97.6 F, blood pressure of 110/65 mmHg, respiratory rate of 18 breaths per minute, heart rate of 109 beats per minute, and oxygen saturation of 99% on room air. On her initial neurologic exam, she was aware of her name, her parents, her birthdate, and where she was. She displayed intermittently slowed speech, which was abnormal for her according to her parents. She had intermittently incomprehensible answers. She had no weakness, no sensory deficits, no cranial nerve deficits, and a normal gait. Initial workup included venous blood gas, blood glucose level, blood lactate level, respiratory pathogen panel, chest radiograph (Figure 3), and head computed tomography (CT) (Figure 4). The patient's laboratory findings are displayed in Table 1.

**Table 1:** Patient's laboratory findings

Laboratory test	Patient’s value	Reference range
Venous pH	7.37	7.32-7.42
Venous pCO2	43.8 mmHg	41.0-51.0 mmHg
Venous HCO3	25.5 mmol/L	24.0-28.0 mmol/L
Venous pO2	36.3 mmHg	25.0-40.0 mmHg
Glucose	164.0 mg/dL	65.0-140.0
Lactate	2.7 mmol/L	0.5-2.0 mmol/L
Respiratory pathogen panel	Negative	Negative

The patient’s chest radiograph was unremarkable for pulmonary injury such as pneumonitis, pneumothorax, or foreign body. The patient's head CT was unremarkable for acute intracranial pathology such as hemorrhage.

The patient was transferred to a tertiary children’s hospital for further care. She was admitted to the hospital medicine service for observation and serial neurological exams. She returned fully to her baseline mental status 12 hours after the injury and was discharged home 24 hours after the injury.

## Discussion

A possible mechanism of injury for this patient is oxygen deprivation from helium inhalation. Helium is colorless and odorless [9]. It has lower density and viscosity than oxygen and, therefore, more easily enters the lungs due to its lower gas resistance [10]. As a result, the inhaled helium quickly displaces inhaled oxygen in the lungs [10]. Symptoms of hypoxia generally begin when environmental oxygen is less than 15-16%, and severe symptoms occur when environmental oxygen is less than 6-8% [11]. Ogden et al. studied the efficacy of oxygen deprivation by helium inhalation in cases of assisted suicide. In the four cases studied, loss of consciousness was rapid, ranging from 36 to 55 seconds [12]. These cases involved inhalation of pure helium gas and involved adult subjects, so no direct comparisons can be made. However, helium has also been demonstrated to be a lethal asphyxiant in children. In a case of homicide, three children were found dead next to balloon gas canisters connected by tubing to plastic bags. The children had postmortem findings consistent with asphyxia, and helium was present in their lung tissues. It was concluded that asphyxiation by helium inhalation was the likely cause of death [13]. Given that the child in this case report was found completely inside a helium-containing environment, it is possible that after entering the balloon, the patient inhaled a large quantity of helium, which displaced the oxygen in her lungs and resulted in loss of consciousness.

Similar incidents have been reported. A 14-year-old died after entering an 86-inch advertising balloon filled with pure helium [14]. His autopsy findings were consistent with suffocation. Helium was present in all his breath samples taken during mechanical ventilation, suggesting that he had inhaled helium gas. The cause of death was determined to be suffocation due to inhalation of atmospheric gas lacking oxygen.

In this case, the patient opened up the balloon along its seam before crawling inside. It is difficult to determine how much helium escaped the balloon prior to the patient’s entrance, so other possible mechanisms of injury must be discussed. An alternate mechanism of injury is mechanical asphyxia from the balloon itself, obstructing the patient’s mouth and nose if she could not extricate herself from the balloon. This type of injury has been described in the literature when describing suicide by plastic bag asphyxiation. Thin, flimsy plastic bags can cling to the mouth and nose during inspiration and cause suffocation by a smothering mechanism [15]. In this case, the foil of the balloon may have similarly clung to her mouth and nose and resulted in smothering.

The patient may also have suffocated inside the balloon after environmental oxygen was consumed during gas exchange, resulting in an oxygen-poor and carbon dioxide-rich environment. Elevated levels of carbon dioxide lead to increased respiratory rate, tachycardia, and impaired consciousness [16]. Very high concentrations of carbon dioxide have been shown in animal models to cause respiratory arrest within one minute [17]. Further research using experimental models would be needed to determine which of these proposed mechanisms of injury is the most likely in this case.

Additional differential diagnoses unrelated to the balloon include seizure, syncope, and arrhythmia. These are less likely primary etiologies for several reasons. First, the patient had no personal or family history of seizures, neurologic disorders, arrhythmias, or sudden cardiac death. Second, although the patient had an episode of urinary incontinence, she had no seizure-like activity. It is possible that a seizure was provoked secondary to the initial injury. Seizures are a known complication of hypoxic-ischemic injury. Seizures secondary to acute hypoxic injury are often secondary to neuronal ischemic necrosis [18]. The patient did not have a brain magnetic resonance imaging study, and subtle neuronal injuries may not have been seen on the patient's CT scan. Still, it would be an improbable coincidence for a primary cardiogenic or neurogenic etiology to initially present when the patient was inside a balloon.

The patient returned to her baseline mental status within 12 hours of the injury. Her normal venous pH and pCO2 and only mildly elevated lactate level were reassuring against severe hypoxic injury and end-organ damage. She showed mild alterations in mental status for several hours after the injury, which could be consistent with mild cerebral hypoxia. It is not known whether the patient had a true pulseless arrest because pulses were not checked before initiating chest compressions. The patient’s oxygen saturation at the time of respiratory arrest is unknown but likely recovered quickly as she was awake and breathing spontaneously on EMS’ arrival.

## Conclusions

Anticipatory guidance on balloons typically revolves around the danger of choking on uninflated balloon fragments. Parents may be familiar with the warning label on packages of latex balloons about the risk of choking or suffocation in children under eight years old. Foil balloons are often considered a safer choice because they don’t typically break into small fragments the way latex balloons do. However, suffocation injuries from large foil balloons are an underrecognized danger. Helium-filled foil balloons are sold in a variety of sizes, including some large enough for even an older child to crawl inside. This case and others reported on in the media highlight that oversized balloons can be enticing yet hazardous hiding spots for children. Parents and pediatricians should be aware of the dangers of suffocation in such cases.
